# Effectiveness of Ultra-Low Volume insecticide spraying to prevent dengue in a non-endemic metropolitan area of Brazil

**DOI:** 10.1371/journal.pcbi.1006831

**Published:** 2019-03-08

**Authors:** Giovanni Marini, Giorgio Guzzetta, Cecilia A. Marques Toledo, Mauro Teixeira, Roberto Rosà, Stefano Merler

**Affiliations:** 1 Dipartimento di Biodiversità ed Ecologia Molecolare, Centro Ricerca e Innovazione, Fondazione Edmund Mach, San Michele all’Adige, Trento, Italy; 2 Epilab-JRU, FEM-FBK Joint Research Unit, Province of Trento, Italy; 3 Center for Information Technology, Bruno Kessler Foundation, Trento, Italy; 4 Departamento de Bioquimica e Imunologia do Instituto de Ciencias Biologicas, Universidade Federal de Minas Gerais, Belo Horizonte, Minas Gerais, Brazil; 5 Center Agriculture Food Environment, University of Trento, San Michele all’Adige (TN), Italy; University of California, Los Angeles, UNITED STATES

## Abstract

Management of vector population is a commonly used method for mitigating transmission of mosquito-borne infections, but quantitative information on its practical public health impact is scarce. We study the effectiveness of Ultra-Low Volume (ULV) insecticide spraying in public spaces for preventing secondary dengue virus (DENV) cases in Porto Alegre, a non-endemic metropolitan area in Brazil. We developed a stochastic transmission model based on detailed entomological, epidemiological and population data, accounting for the geographical distribution of mosquitoes and humans in the study area and spatial transmission dynamics. The model was calibrated against the distribution of DENV cluster sizes previously estimated from the same geographical setting. We estimated a ULV-induced mortality of 40% for mosquitoes and found that the implemented control protocol avoided about 24% of symptomatic cases occurred in the area throughout the 2015–2016 epidemic season. Increasing the radius of treatment or the mortality of mosquitoes by treating gardens and/or indoor premises would greatly improve the result of control, but trade-offs with respect to increased efforts need to be carefully analyzed. We found a moderate effectiveness for ULV-spraying in public areas, mainly due to the limited ability of this strategy in effectively controlling the vector population. These results can be used to support the design of control strategies in low-incidence, non-endemic settings.

## Author summary

Dengue is a mosquito-borne infection that causes millions of symptomatic infections and thousands of deaths per year. This pathogen is expanding its geographic range to areas that were previously free from autochthonous transmission thanks to the intensification of international travels, urbanization and to climatic adaptation of mosquitoes and viruses. Usually interventions against dengue transmission consist in insecticide spraying aimed at killing adult mosquitoes, but the impact of this practice has been rarely evaluated in real-life settings. In this work, we estimate the proportion of dengue cases avoided by Ultra-Low-Volume insecticide spraying in public areas in Porto Alegre (Brazil). This city is characterized by a subtropical climate, negligible pre-existing immunity and low dengue incidence. The low incidence makes this region unsuitable for deployment of the currently licensed vaccine, which is only recommended by the WHO for high-transmission areas. We found that insecticide spraying avoided approximately one fourth of all symptomatic cases. The performance of the intervention was negatively affected by the low treatment-induced mosquito mortality, as we estimated that only 40% of *Ae*. *aegypti* are killed by the insecticide. Control outcomes could be improved by increasing the targeted area and including private premises, but trade-offs against increased efforts need to be carefully analyzed.

## Introduction

Dengue virus (DENV) causes a considerable burden to public health worldwide, consisting of 60 to 100 million symptomatic infections, 14,000 to 20,000 deaths per year, and of a global annual cost of about 9 billion dollars [1, 2]. Four distinct DENV serotypes exist; infection by one of them confers life-long immunity to that serotype and temporary cross-immunity to others. However, secondary dengue infections with different serotypes are more likely to cause severe illness because of an immune process known as antibody-dependent enhancement (ADE), where pre-existing cross-reacting antibodies do not neutralize but rather enhance viral replication [3].

In Southern America, continental control efforts in the 1970s had come close to eliminate *Aedes aegypti*, the main vector mosquito, but in the last two decades arboviruses have strongly increased their circulation [4] thanks to the intensification of international travels [5], international trade [6] and urbanization [7] and to climatic adaptation of mosquitoes and viruses [8]. In particular, DENV is expanding its geographic range to areas that were previously free from autochthonous transmission and are now prone to multiple outbreaks each year. A DENV vaccine has been recently licensed, with an overall efficacy of about 60% against its four serotypes [9]; however, after deployment in 10 different countries, increased hospitalization rates in children were reported, raising suspects that the currently available vaccine may predispose individuals who were seronegative before vaccination to ADE [10]. Therefore, modeling studies and the World Health Organization recommend the vaccine for high-transmission settings only where the proportion of seronegative vaccinees is very low [11, 12]. In non-endemic settings, the main strategy for DENV control consists in the management of vector populations via insecticide spraying as a reactive intervention to ongoing local transmission. Despite its popularity, this practice has been rarely evaluated in terms of its impact on DENV transmission in real-life settings [13, 14]. Such evaluation is made difficult by the inherently complex interactions between dynamics of mosquito populations, viral transmission and insecticide uptake in structured spaces and time-varying environmental conditions [15]. Furthermore, DENV transmission and control interventions typically occur over geographic scales of a few hundred meters [16–18], making it necessary to consider spatial heterogeneities explicitly and at a high resolution [19]. To estimate the effectiveness of insecticide spraying on DENV containment, we developed a novel mechanistic computational framework, coupling a model for mosquito population dynamics and a disease transmission model over a high-resolution spatial grid. Mechanistic models are widely used to study mosquito-borne pathogens such as DENV, for instance to investigate outbreaks in a previously unaffected area [20], assess vaccine effectiveness [11, 21, 22] or to compare transmission of different viruses in the same area [23]. Many of the available models do not include explicitly the mosquito immature stages [24], which need to be considered to accurately reproduce the dynamics of recovery of the vector population after adulticide treatment. Spatial transmission models are another common epidemiological tool that has been used to study DENV control in different parts of the world [21, 22, 25–27]. Here, we combine these approaches with recent insights on the spatiotemporal dynamics of DENV [16], to estimate the proportion of cases avoided by Ultra-Low-Volume (ULV) insecticide spraying in Porto Alegre, a Brazilian metropolis characterized by a subtropical climate, low DENV incidence and negligible pre-existing immunity.

## Materials and methods

### Study area

Porto Alegre (30°01′40″S, 51°13′43″W) is the capital of Rio Grande do Sul, the southernmost state of Brazil, with a population of 1,400,000 inhabitants [28] spread over an area of 500 km^2^ and characterized by a subtropical humid climate (Cfa) [29]. Local transmission of DENV has been recorded only since 2010 [30] and epidemics with more than 1,000 suspected cases have been recorded in 2013 and 2016. In these years, the effective reproduction number exceeded the epidemic threshold between January and mid-March, with a peak value for of 1.5 [16]. Over 70% of all transmission events were estimated to occur within 500m from the residence of the infector [16].

To face the emergence of DENV, an integrated surveillance and prevention protocol has been put in place that includes entomological, virological, and active epidemiological components [31]. The reactive control policies implemented to reduce DENV transmission consisted in ULV insecticide spraying in public spaces such as roads and parks (i.e., not indoor or in private gardens), within an area of radius 200m around the residence of the patient triggering the intervention. The decision to treat an area was made depending on lab-confirmation of a case through ELISA immunological tests, previous treatment in the same area, and availability of resources at the time of decision. Table 1 reports the resulting observed frequency of treatment initiation for imported (*q*_*I*_) and locally transmitted (*q*_*A*_) cases, as classified by epidemiological investigations. Treatments were not systematic for confirmed cases because of limited resources, especially in cases when further transmission was not considered to be likely by public authorities, e.g. when a treatment had already been performed in the previous days in the same area.

**Table 1:** Model parameters.

Para-meter	Description	Value	Source
*p*_*I*_	Proportion of imported symptomatic cases that were confirmed	0.47	Notification data
*p*_*A*_	Proportion of autochthonous symptomatic cases that were confirmed	0.27	Notification data
*q*_*I*_	Proportion of treatment initiations after confirmation of an imported symptomatic case	0.6	Treatment data
*q*_*A*_	Proportion of treatment initiations after confirmation of an autochthonous symptomatic case	0.46	Treatment data
$\bar{\Omega }$	Average number of secondary symptomatic infections caused by symptomatic index cases	10.6 (95%CI: 6.7–14.5)	[16]
η	Rate of exponential decrease in the force of infection	0.015 m^-1^	[16]
*r*	Radius of ULV-treated area	Baseline: 200 meters	Treatment protocol
τ	Delay between symptoms onset of the triggering case and date of ULV treatment	N($\bar{\tau }$, 8) days, Baseline: $\bar{\tau }$ = 14	Treatment data
*Ψ*	Scaling factor for the force of infection	0.57	Calibrated
ρ	Baseline vector control efficacy	Baseline: 40%	Calibrated

### Data

**Fig 1:**
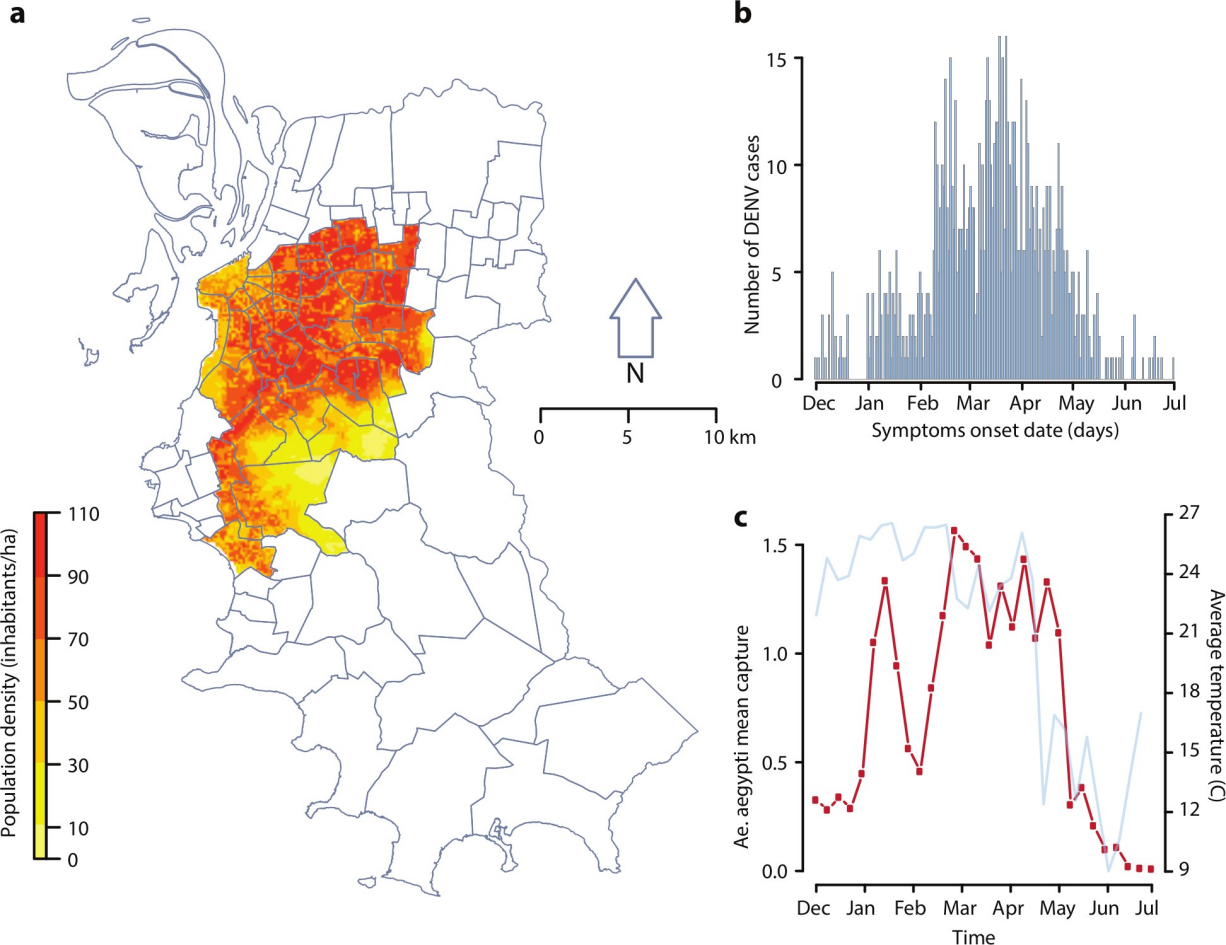
Study area, DENV recorded cases, mosquito collections and temperature data. a) Map of the selected study area within the city of Porto Alegre and corresponding population density; b) dengue cases in the selected study area over time; c) average captures of *Ae*. *aegypti* female adults per trap over time (red, left axis), with corresponding weekly temperature values (light blue, right axis). Map was generated using shapefiles publicly available from Porto Alegre municipality.

We considered DENV transmission in 42 central neighborhoods of Porto Alegre, over the period between December 1, 2015 and June 30, 2016. We used human population data from the Worldpop database [32] spatially disaggregated at 100m resolution over the considered study area (99.2 km^2^, about 20% of the total city area). The total population was 605,260 inhabitants (43% of the total city population), with human density values ranging from 5.3 to 102.5 persons per hectare (Fig 1A). Temperature data for the whole city were obtained from the Brazilian National Institute of Meteorology (INMET) [33]. The mean daily temperature for the study period ranged between 7.4 and 29.5°C, with an average of 21.1°C (Fig 1C).

Data on female adult *Ae*. *aegypti* mosquitoes were previously collected using MosquiTRAP (Ecovec LTDA, Belo Horizonte, Brazil) sticky traps [34] containing a synthetic oviposition attractant (AtrAedes) that lures gravid *Ae*. *aegypti*. Overall, 776 geolocalized sticky traps were set on fixed outdoor positions at a distance of 250m between each other and inspected weekly (Fig 1C). The Health Secretary of Porto Alegre provided notification data for autochthonous and imported dengue cases. 891 suspected cases with symptom onset between December 2015 and June 2016 were reported in the study area (Fig 1B).

### Computational framework

**Fig 2:**
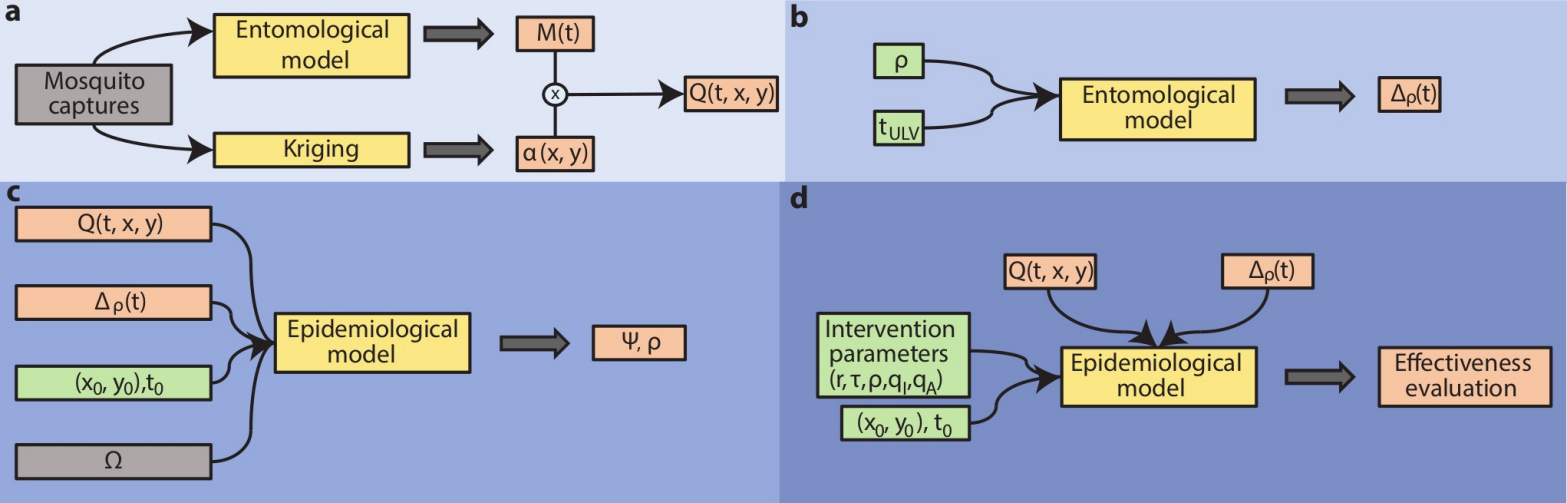
Schematic representation of the computational framework. a) Estimation of the daily mosquito abundance for each geographic cell; b) modelling the effect of ULV treatments on mosquito dynamics; c) epidemiological model calibration; d) estimation of ULV treatments effectiveness under different protocols. Grey: observed data; yellow: models; green: model parameters; red: model estimates. See text for a complete description of symbols and models.

Fig 2 shows a schematic representation of the computational framework adopted in this analysis. *Aedes aegypti* capture data were used to estimate the spatiotemporal distribution of mosquito abundance in the study area and period (Fig 2A). Data were aggregated across all traps to obtain an overall time series of *Ae*. *aegypti* captures in Porto Alegre (Fig 1C), which was used to calibrate a mosquito population model (“entomological model”) and provide an estimation of the daily total mosquito abundance *M*(*t*). The entomological model adapts a previously published approach [35] representing the biology of *Ae*. *aegypti* life stages via temperature-dependent parameters. Larval carrying capacity was calibrated to weekly capture data with a Monte Carlo Markov Chain approach.

For each trap, the total number of captures over time was used as an estimate of the local abundance of mosquitoes; these estimates were interpolated throughout the study area via standard kriging techniques, obtaining the relative abundance of mosquitoes, *α(x*, *y)*, over a spatial grid of 9919 geographic cells (100m x 100m) covering the study area.

The mosquito abundance over time and space, *Q*(*t*, *x*, *y*), was finally obtained via the combination of these two estimates:

Q(t,x,y)=M(t)⋅α(x,y).

The calibrated entomological model was used to mechanistically simulate the effect of ULV spraying and the ensuing recovery of mosquito populations (Fig 2B).

Given a time of intervention *t*_*ULV*_ and a mosquito-induced mortality ρ, we computed the relative reduction Δ_ρ_(*t*) of the mosquito abundance after treatment *M*_*ULV*_(ρ,*t*), compared to the baseline:

$$\Delta_{\rho }(t)=\left\{ \begin{array}{l} \frac{M(t)-M_{ULV}(\rho ,t)}{M(t)},\;t\geq t_{ULV}\\ \;0\;,\;t<t_{ULV} \end{array}\right.$$

When a treatment is initiated in any cell *i*, the mosquito abundance of all cells within a distance *r* (the radius of the ULV-treated area) from *i* is rescaled by a factor 1-Δ_ρ_(*t*). This equation allows us to consider that: i) ULV treatment causes the sudden death of a proportion ρ of adult mosquitoes; ii) following ULV spraying, the mosquito population recovers as new adults emerge from pre-existing immature stages (which are not affected by adulticides), as well as from newly deposed eggs from surviving mosquitoes, as predicted by the entomological model.

The spatiotemporal DENV dynamics was implemented by considering a standard SEIR-SEI epidemiological model [11, 20, 23] where human-to-vector and vector-to-human virus transmission is regulated by temperature-dependent parameters and can occur across cells via a previously estimated distance-dependent kernel [16] (Fig 2C). Given the negligible pre-existing immunity (only one positive individuals over 422 tested was found in a seroprevalence study in 2015; C. Marquez Toledo, personal communication, November 2017) and cross-serotype ecological interactions, we modelled infection independently of DENV serotype and we assumed that the human population was fully susceptible to the infection. Asymptomatic individuals were assumed to be unable to transmit the virus; however, in a sensitivity analysis we allowed for asymptomatic transmission with different rates [11].

The epidemiological model is applied to simulate single transmission clusters originating from one infectious individual imported at coordinates (*x*_0_, *y*_0_) and time *t*_0_. A transmission cluster is defined as the set of all human infections directly and indirectly generated by the index case until stochastic fadeout of the chain of transmission [36] (i.e. when the number of exposed and infectious mosquitoes and humans is zero) and the cluster size is defined as the number of secondary symptomatic infections [16].

We modeled interventions according to implemented control protocols: each symptomatic case had a probability of being lab-confirmed and, in such case, a probability to trigger vector control interventions after a delay since symptom onset τ, sampled from a Normal distribution, truncated to positive values. To reflect control protocols implemented in Porto Alegre, we assigned different probabilities of treatment to imported and autochthonous cases.

DENV transmissibility in the model, mediated by parameter *ψ*, and the ULV-induced mosquito mortality, ρ, were calibrated to reproduce the size distribution (average $\bar{\Omega }$) of the 76 clusters occurring in the study area and period [16].

Finally, the calibrated computational framework was used to evaluate the effectiveness of ULV spraying by comparing the number of symptomatic DENV cases obtained with and without treatment (Fig 2D). In addition, to evaluate the effect of different control protocols, we explored the impact of different values of *r*, τ and ρ on the relative reduction of DENV cases.

To ensure the robustness of results, we simulated for each considered scenario 20,000 transmission clusters by sampling the index case’s coordinates (with probability proportional to the local population density) and time (uniformly between December 1, 2015 and April 30, 2016). For clusters with at least one secondary transmission, the cluster duration was defined as the number of days necessary for stochastic fadeout since symptom onset of the index case, and the cluster radius as the maximal distance between the location of the index case and all other cases in the cluster.

Full details on implementation of different components of the modeling framework are reported in the S1 Appendix; parameter values for the epidemiological model are reported in Table 1. For all estimates, we computed 95% confidence intervals of the average using the Student’s t-test.

## Results

**Fig 3:**
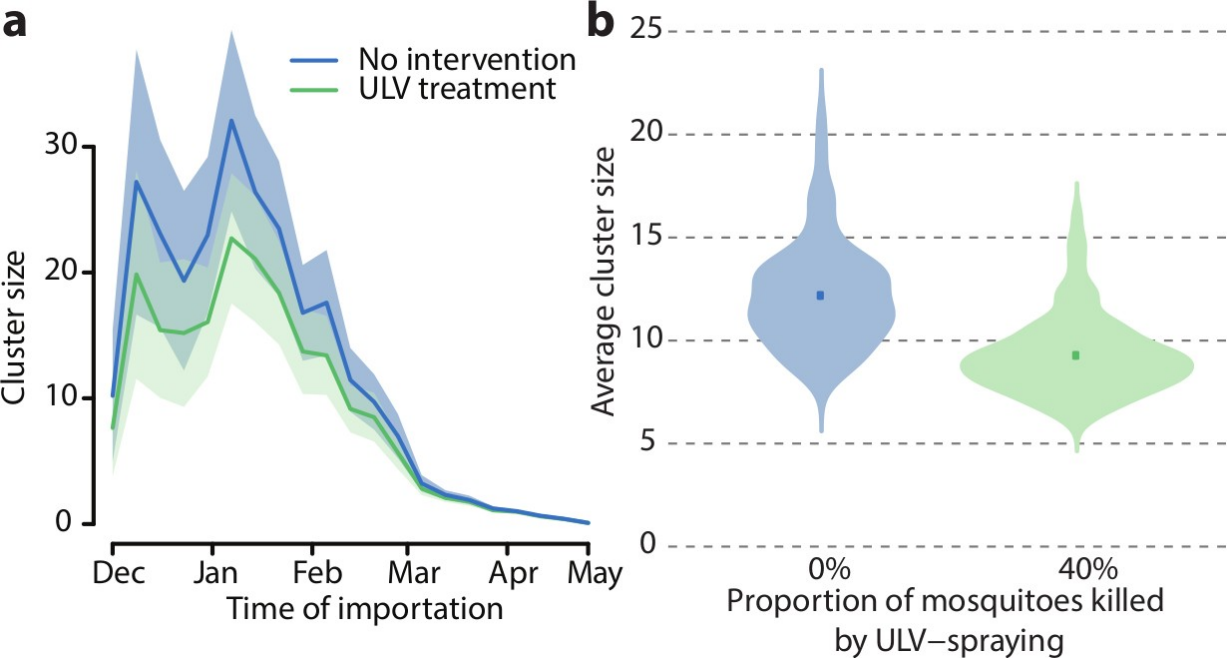
Effect of control interventions on cluster size. a) Cluster size over time. Solid lines: average; shaded area: 95% credible interval. b) Violin plots of the distribution of the average cluster size with and without control; the distributions are computed from 100 averages over 1,000 clusters randomly sampled from the 20,000 simulated clusters. Squares represent the average values.

In order to reproduce the observed distribution of cluster sizes in Porto Alegre [16], the mortality of mosquitoes due to ULV treatment in public spaces was estimated at about 40% (see S1 Appendix), which is in good agreement with previous experimental estimates [37–40]. In absence of control interventions, our model estimated that the 76 clusters observed in the study area and period would have caused 1055 secondary symptomatic cases (95%CI: 995–1113), which, compared to the observed 815, implies that 240 cases (95%CI: 180–298) were avoided by the implemented protocols. The simulated average cluster size in absence of intervention was 11.9 (95%CI: 10.9–12.9) cases, with a peak of 32.1 (95%CI: 24.9–39.3) for importations occurring in the second week of January, falling to less than one for cases imported at the end of April (Fig 3A). ULV treatment was able to moderately reduce the average cluster size to 9.1 (95%CI: 8.3–9.8), i.e. by 23.9% (95% CI: 17.5–30.2%) (Fig 3B); the difference in cluster size distributions was significant according to a Wilcoxon-Mann-Whitney test p-value<0.001. Treatment was most effective during the months of highest transmissibility, with a peak reduction of symptomatic cases by 38.0% (95%CI: 36.8–40.4%) for clusters initiated at the end of December.

**Fig 4:**
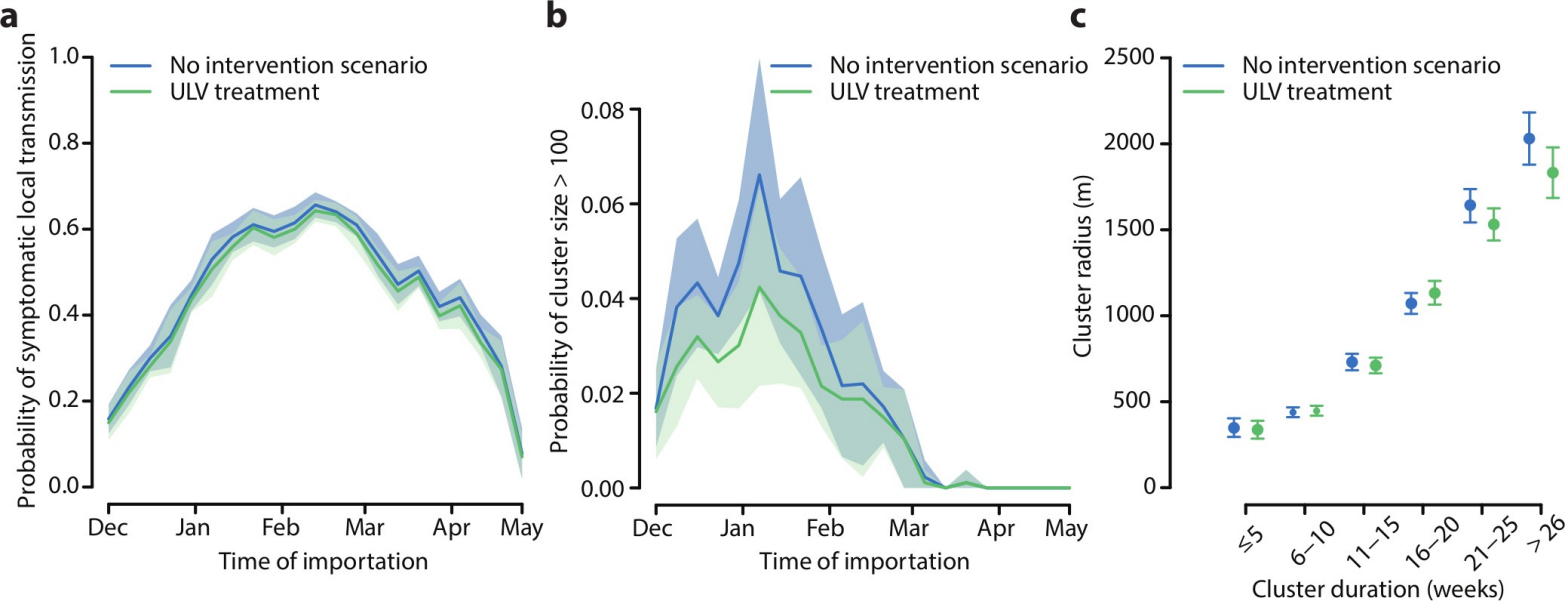
Effects of ULV-spraying. a) Probability that an imported case will cause secondary symptomatic cases. b) Probability of cluster size greater than 100. c) Relation between cluster duration and cluster radius (excluding clusters with no secondary symptomatic cases). Solid lines: average; shaded area: 95% credible interval.

Treatment reduced only marginally the average probability of symptomatic local transmission, i.e. the probability that an imported case caused at least one secondary symptomatic case. The estimated probability of symptomatic local transmission from our model, including treatments, was similar to previous estimates in Porto Alegre for 2016 [16] and had a peak of 64% (95%CI: 62–67%) at the end of February (Fig 4A). On the other hand, the probability of clusters of size larger than 100 was more markedly reduced by treatment (from 2.1% on average in the case of no intervention, to 1.6%, Fig 4B). Treatment was responsible of an almost negligible reduction of the average outbreak duration (from 13.6 weeks, 95%CI: 13.5–13.7, without ULV spraying, to 13.1 weeks, 95%CI: 12.9–13.2) and of the average cluster radius (from 853m, 95%CI 827-878m, to 806m, 95%CI: 781-831m, see Fig 4C). Outbreak duration and cluster radius were strongly correlated (Spearman correlation coefficient 0.62, p-value<0.001, both with and without treatment).

**Fig 5:**
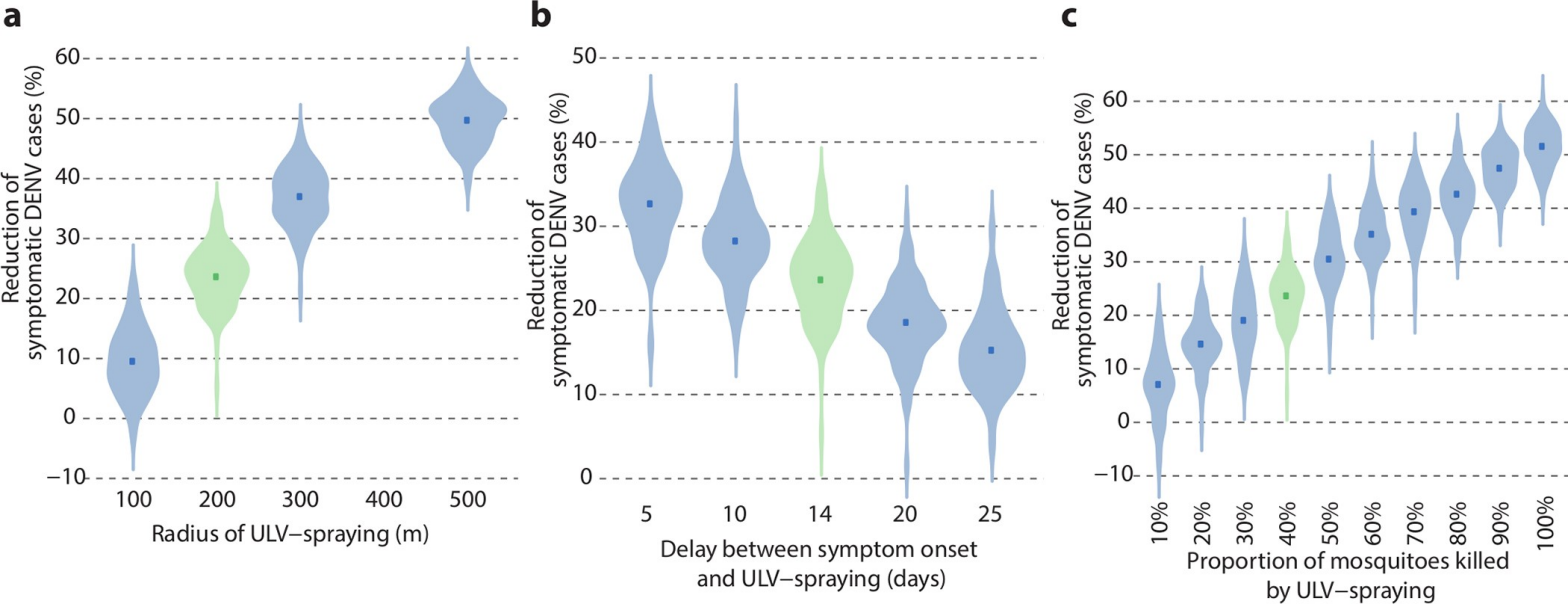
Sensitivity analysis on effectiveness of control interventions. Violin plots for the distributions of the average percentage of avoided DENV symptomatic cases by (a) radius of the treated area, (b) average delay between symptom onset and intervention and (c) proportion of mosquitoes killed by ULV spraying. The distributions are computed from 100 averages over 1,000 clusters randomly sampled from the 20,000 simulated clusters. Squares represent the average values.

We found that the proportion of avoided DENV cases changes significantly when varying the radius of the treated area (see Fig 5A): from 10.6% (95%CI: 3.1–18.0%) with a radius of 100m to 37.1% (95%CI: 32–42.2%) for a radius of 300m and 50.0% (95%CI: 46.4–53.6%) with a radius of 500m. Treatment effectiveness improved when reducing the average delay after symptom onset to 5 days (Fig 5B), with a proportion of avoided cases of 32.8% (95%CI: 27.2–38.3%), while increasing the average delay to 25 days would reduce the average effectiveness to 16.1% (95%CI: 9–23.2%). Changes in the proportion of mosquitoes killed by ULV resulted in proportional reductions of secondary symptomatic DENV cases, up to an average maximum of 52% (95%CI: 48.2–55.7%) when all existing mosquitoes are killed by treatment under current intervention protocols (Fig 5C).

Our estimates are robust with respect to the assumption on the relative transmissibility of DENV by asymptomatic individuals. In scenarios where asymptomatic individuals have a transmission rate of 50% and 100% compared to symptomatic patients [11], the estimated average relative reduction of cases, assuming the same ULV induced mosquito mortality (ρ = 40%), was 23.3% (95%CI: 15.1–31.4%) and 22.1% (95%CI: 13.9–30.2%) respectively (see S1 Appendix).

## Discussion

Using a spatial stochastic model informed with geolocated capture data on *Ae*. *aegypti* female adults and with previous estimates on the size of dengue clusters in Porto Alegre, we estimated the effectiveness of implemented control interventions in terms of the reduction of symptomatic dengue cases. To the best of our knowledge, the impact of ULV outdoor spraying on the reduction of DENV transmissibility has never been estimated using observational data in non-endemic areas [13]. A theoretical assessment of different containment procedures including adulticide spraying has been previously suggested [26], while others [25, 27] evaluated indoor spraying effectiveness in regions with high DENV circulation. We found that ULV insecticide spraying in public places avoided approximately one fourth of all secondary symptomatic DENV cases, corresponding to roughly 240 cases in an area of about 100km^2^ over a full epidemic year. The performance of the intervention was negatively affected by the low estimated efficiency in killing existing mosquitoes in the treated area (about 40%). This value is compatible with field experiments showing that the majority of *Ae*. *aegypti* rest within households [37]; furthermore, other studies measured the mortality of mosquitoes resting in sheltered locations [38] or in the vegetation [39, 40] at values between 30% and 50%, indicating a moderate effect of ULV treatment on mosquito populations.

To improve the effectiveness of control, a larger area might be treated, but the trade-off between increasing effort and increasing benefits needs to be taken into account. We estimate that increasing the intervention radius to 300m around the triggering case would result in about 143 additionally avoided cases (an increase by 60%) compared to the current protocol but requires treating a total area that is 2.25 times larger. An alternative way to improve the current control strategy would be to target private gardens and/or indoor spaces. An increase in the proportion of killed mosquitoes from 40% to 60–70% would improve the overall effectiveness by a similar amount than that allowed by expanding the treated radius to 300m. A similar efficacy could also be achieved by increasing the frequency of treatment for confirmed cases from 60% (*q*_*I*_) and 46% (*q*_*A*_) to 100%, which would avoid 38.6% (95%CI: 33.6–43.7%, see S1 Appendix) of the expected cases. Alternative approaches towards the reduction of DENV transmission might consider routine preventive interventions, rather than reactive ones, such as deploying larvicides in city areas with highest mosquito abundance [41].

A potential source of uncertainty in our study is the role of asymptomatic individuals in viral transmission. Duong et al. have shown that mosquitoes can be infected by asymptomatic and pre-symptomatic children [42], but key parameters such as their transmission rate to mosquitoes, the transmission rate to humans by mosquitoes infected by asymptomatic individuals and the asymptomatic infectious period, remain unknown. Since the study by Duong et al., asymptomatic transmission has been included in most recent transmission models for dengue as a reduced human-to-mosquito transmission rate arbitrarily fixed between 0% and 50% of the value for symptomatic individuals [11, 23]. Here, we assumed as a baseline that asymptomatic individuals do not transmit the infection; however, we run two alternative scenarios where asymptomatic transmission occurred as the symptomatic one and also at a relative transmission rate of 50% and we found that in both cases the relative reduction of DENV cases granted by ULV treatment would be similar (see S1 Appendix).

Among further potential limitations, we acknowledge that large temperature fluctuations may have a negative impact on *Ae*. *aegypti* biology [43]. However, in the absence of sufficient data to parametrize these effects, we modelled mosquito population dynamics by considering only average daily temperatures, similarly to previously published modelling studies (e.g. [20, 25]). Furthermore, our temperature data came from a single weather station for the whole city. The effect of within-day and local variability of temperature may be a source of bias for our estimates, whose impact is very difficult to assess.

We did not consider the potential effect of treatment on mosquito capture data for the estimation of the mosquito population. This assumption was based on two observations: first, the limited capture rate of sticky traps, combined with the coarse (weekly) temporal resolution of captures and the small ULV-induced mosquito mortality, make it difficult to detect a significant effect of treatment on capture data (see S1 Appendix for details); second, treatment at any given time included a very limited proportion of the study area, so that the expected effect on the total abundance in the area is marginal. As a proof, we re-computed the mosquito abundance by simulating treatments at the time and sites where they were actually administered during 2016, assuming an effectiveness ρ = 40%; the maximum difference in the total mosquito population compared to the no-treatment scenario was less than 10% at all times.

In the absence of a safe vaccine for DENV in non-endemic settings, the prevention of dengue transmission will continue to rely on the management of mosquito populations. Quantifying how ULV mosquito control translates into a mitigation of the disease burden is a critical but still unanswered public health question [13, 14]. This study provides a quantitative estimate of the effectiveness of ULV treatment in a non-endemic setting where dengue transmission has established only recently, based on recent insights on the spatiotemporal dynamics of dengue and on high-resolution entomological, population, clinical and treatment data. Our results can be used to support the design and implementation of future interventions in areas at the margins of the geographical range of DENV (e.g. in Southern Europe, USA, subtropical South America and Australia) which are undergoing a similar epidemiological transition, or are expected to do so in the next future.

## Supporting information

S1 Appendix Supporting text containing methodological details and additional results. (PDF)

S1 Table Entomological dataset. (XLSX)
