# Supplementary material for: Effectiveness of Ultra-Low Volume insecticide spraying to prevent dengue in a non-endemic metropolitan area of Brazil
Source: PLoS Comput Biol. 2019 Mar 8;15(3):e1006831. doi: 10.1371/journal.pcbi.1006831 (PMC6426269; doi:10.1371/journal.pcbi.1006831)
Supplement: S1 Appendix — (PDF) [file pcbi.1006831.s001.pdf]

Effectiveness of Ultra-Low Volume insecticide spraying to prevent dengue in a non-endemic metropolitan area of Brazil

Giovanni Marini[1], Giorgio Guzzetta[2,3], Cecilia A. Marques Toledo[4], Mauro Teixeira[4], Roberto Rosà[1,2,5*], Stefano Merler[2,3]

1) Dipartimento di Biodiversità ed Ecologia Molecolare, Centro Ricerca e Innovazione, Fondazione Edmund Mach, via E. Mach 1, I-38010 San Michele all'Adige, Trento, Italy;

2) Epilab-JRU, FEM-FBK Joint Research Unit, Province of Trento, Italy;

3) Center for Information Technology, Bruno Kessler Foundation, via Sommarive, 18 I-38123 Trento, Italy;

4) Departamento de Bioquimica e Imunologia do Instituto de Ciencias Biologicas, Universidade Federal de Minas Gerais, Av. Antônio Carlos, 6627 - Pampulha CEP: 31270-901, Belo Horizonte, Minas Gerais, Brazil.

5) Center Agriculture Food Environment, University of Trento, via E. Mach 1, I-38010, S. Michele all'Adige (TN), Italy

* Corresponding author: roberto.rosa@fmach.it

Appendix

Table of contents

Methods 2

Entomological model 2

Vector control 6

Spatial DENV transmission model 7

Supplementary results 10

Effect of treatment on mosquito capture data 10

Full results from sensitivity analyses 10

Transmission by asymptomatic individuals 12

Systematic treatment of cases 14

References 15

Methods

Entomological model

The population dynamics of *Ae. aegypti* is modelled adapting the approach proposed by (Otero et al. 2006). The model accounts for 5 life stages, namely eggs (E), larvae (L), pupae (P), adult females in their first gonotrophic cycle, (A_1), and in subsequent gonotrophic cycles (A_2), plus a compartment (C) representing captured mosquitoes. The time-continuous version of the model is based on the following system of Ordinary Differential Equations (ODE):

$$
\begin{aligned}
E' &= n_E\left(d_{A_1}A_1 + d_{A_2}A_2\right) - (\mu_E + d_E)E \\
L' &= d_E E - \mu_L\left(1 + \frac{L}{K}\right)L - d_L L \\
P' &= d_L L - (\mu_P + d_P)P \\
A_1' &= \frac{1}{2}e_P d_P P - \left(\mu_A + d_{A_1} + d_{A_1}\beta\right)A_1 \\
A_2' &= d_{A_1}A_1 - \left(\mu_A + d_{A_2}\beta\right)A_2 \\
C' &= d_{A_1}\beta A_1 + d_{A_2}\beta A_2
\end{aligned}
$$

where

- d_{A_1}, d_E, d_L, d_P are temperature-dependent developmental rates driving the vector transitions across different life stages;
- $\mu_E, \mu_L, \mu_P, \mu_A$ are temperature-dependent death rates associated with the different stages;
- n_E is the average number of eggs laid in each oviposition by a single adult female;
- d_{A_1} and d_{A_2} are the oviposition rates (given by the inverse of the average length of gonotrophic cycle for the two adult stages);
- K is a density-dependent scaling factor driving the carrying capacity for the larval stage;
- e_P is the emergence success factor, i.e. the probability that an emerging pupa becomes an adult and does not die during the process;
- β is the daily adult capture rate;
- C represents the cumulative number of captured female adult mosquitoes; its state equation depends on the gonotrophic cycle because the traps used are only attractive for gravid mosquitoes.

Since only female adult mosquitoes are explicitly considered in the model, the term 1/2 in the equation for the adults (A_1) accounts for the sex ratio (Otero et al. 2006).

We implemented model M as a time-discrete deterministic version, with time step $\Delta t = 1$ day. The seasonal dynamics of the mosquito population is simulated from December 1, 2015 to June 30, 2016.

Values and the functional dependence on temperature for mosquito life-cycle parameters are reported in Table A and derived from (Otero et al. 2006). The capture rate β was estimated at $7.5 \cdot 10^{-5}$/day from a mark-release-recapture experiment (Maciel-de-Freitas & Loureno-de-Oliveira 2009) while the larval scaling factor K was calibrated by fitting capture data with a Monte Carlo Markov Chain approach. Specifically, we assumed that, for each capture session, the number of captured female adult mosquitoes follows a Poisson distribution with mean obtained from the model; therefore the likelihood of the observed data given K has been defined as

$$L = \prod_{i=1}^{h} e^{\{-\tilde{C}_i(K)\}} \frac{\tilde{C}_i(K)^{\,C_i}}{C_i!}$$

where i runs over the number of capture sessions h, C_i is the observed number of captured *Ae. aegypti* adults at capture session i and $\tilde{C}_i(K)$ is the predicted number of captures at capture session i simulated by the model with parameter K.

As reported in Figure A, the model reproduces well the overall seasonal dynamics, although it is not able to replicate a sudden abundance drop in captured mosquitoes occurring in February.

**Parameter**	**Explanation**	**Value/Function**
n_E	Number of eggs laid by a gravid female	63
d_{A1}	First oviposition rate	$0.216 \cdot \frac{T_k}{298} \cdot \frac{\exp\left(\frac{15725}{1.987} \cdot \left(\frac{1}{298} - \frac{1}{T_k}\right)\right)}{1 + \exp\left(\frac{1756481}{1.987} \cdot \left(\frac{1}{447.2} - \frac{1}{T_k}\right)\right)}$
d_{A2}	Subsequent ovipositions rate	$0.372 \cdot \frac{T_k}{298} \cdot \frac{\exp\left(\frac{15725}{1.987} \cdot \left(\frac{1}{298} - \frac{1}{T_k}\right)\right)}{1 + \exp\left(\frac{1756481}{1.987} \cdot \left(\frac{1}{447.2} - \frac{1}{T_k}\right)\right)}$
d_E	Egg hatching rate	$0.24 \cdot \frac{T_k}{298} \cdot \frac{\exp\left(\frac{10798}{1.987} \cdot \left(\frac{1}{298} - \frac{1}{T_k}\right)\right)}{1 + \exp\left(\frac{100000}{1.987} \cdot \left(\frac{1}{14184} - \frac{1}{T_k}\right)\right)}$
d_L	Larval development rate	$0.2088 \cdot \frac{T_k}{298} \cdot \frac{\exp\left(\frac{26018}{1.987} \cdot \left(\frac{1}{298} - \frac{1}{T_k}\right)\right)}{1 + \exp\left(\frac{55990}{1.987} \cdot \left(\frac{1}{304.6} - \frac{1}{T_k}\right)\right)}$
d_P	Pupal development rate	$0.384 \cdot \frac{T_k}{298} \cdot \frac{\exp\left(\frac{14931}{1.987} \cdot \left(\frac{1}{298} - \frac{1}{T_k}\right)\right)}{1 + \exp\left(-\frac{472379}{1.987} \cdot \left(\frac{1}{148} - \frac{1}{T_k}\right)\right)}$
μ_E	Egg death rate	0.01
μ_L	Larva death rate	$0.01 + 0.9725 \cdot \exp\left(-\frac{(T_k - 278)}{2.7035}\right)$
μ_P	Pupa death rate	$0.01 + 0.9725 \cdot \exp\left(-\frac{(T_k - 278)}{2.7035}\right)$
e_P	Pupa emergence success factor	0.83
μ_A	Adult death rate	0.09

Table A. Parameters of the entomological model. T_K denotes the temperature in Kelvin. The source for all values and functions is (Otero et al. 2006).

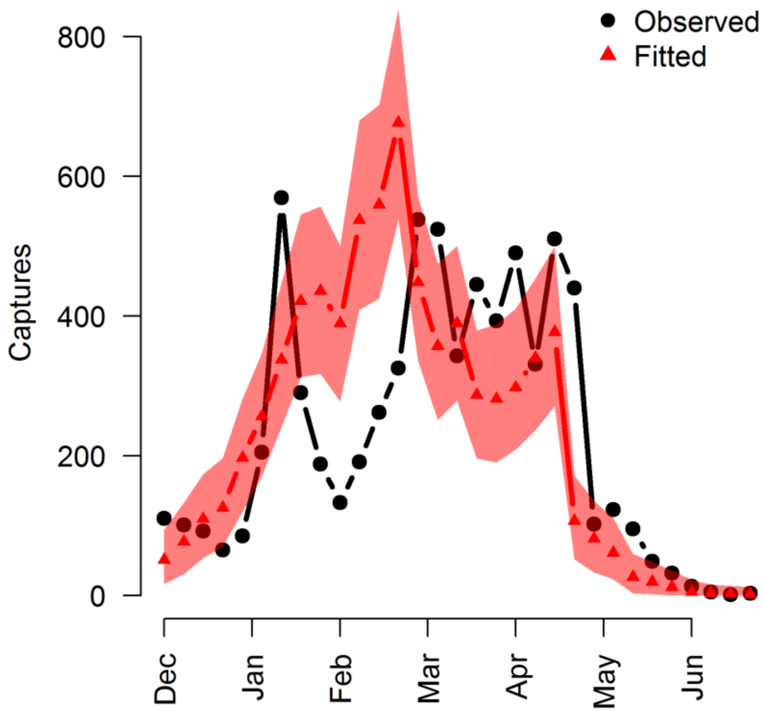

Figure A. *Ae. aegypti* model fit. Observed total captures (black dots) and model prediction (red triangles: average; shaded area: 95% credible interval).

The daily abundance of adult females $M(t)=(A_1(t)+A_2(t))$ predicted for the whole study area was spatially redistributed based on the total yearly captures of mosquitoes in geolocated traps, using a standard kriging, obtaining an estimate of vector abundance for each cell i, indicated by coordinates (x, y), and each day t, denoted with $Q(t, x, y)$. To summarize, we fitted the variogram for each trap location according to its total captures, which allowed us to give a normalized weight $\alpha(x, y)$ to each pixel i of the study area and thus estimate $Q(t, x, y)=\alpha(x, y)\cdot(A_1(t)+A_2(t))$. Figure B shows the estimated spatially distributed abundances for the first day of the first four months (December-March) of our study period. *Aedes aegypti* was heterogeneously distributed over the city of Porto Alegre, with higher densities in the northern part of the selected study area. The cell-specific mosquito daily density (number of mosquitoes per hectare) during the study period (December 1, 2015-June 30, 2016) ranges between 58 and 19,712, with an average of 5,692.

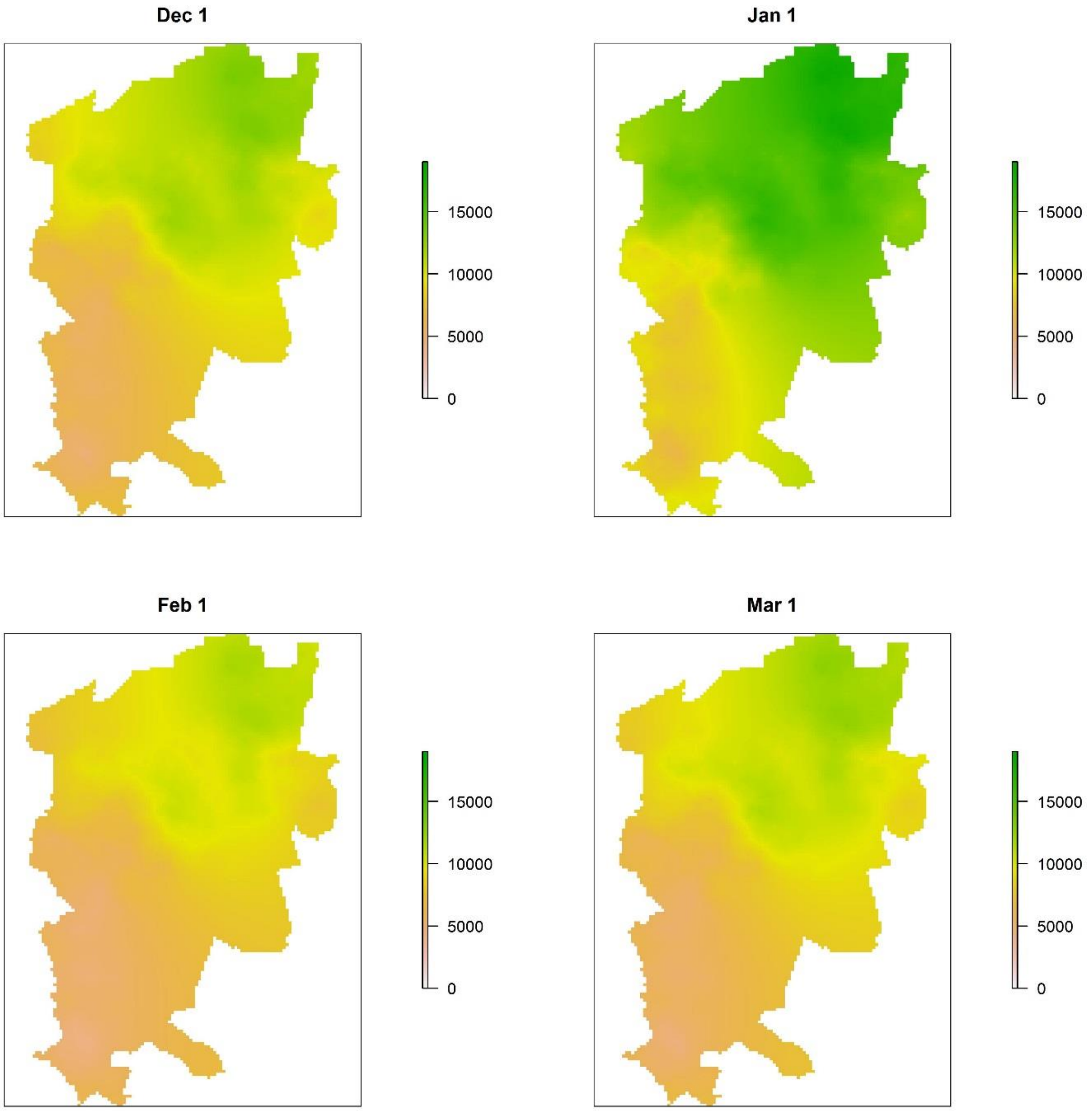

Figure B. *Ae. aegypti* estimated density (number of adult mosquitoes per hectare). Maps were generated using shapefiles publicly available from Porto Alegre municipality.

By comparing Figure B and Figure 1a in the main text, we can note that highly human populated areas are also very suitable habitats for *Ae. aegypti* (Spearman correlation coefficient between human and mosquito densities: 0.46, p-value<0.001), confirming that this is a highly domesticated mosquito species that prefers to live in urban environments, to feed on humans and to lay eggs in artificial containers (Gubler 2011).

Vector control

We used our mosquito model to explore the effects of ULV-treatment over time, i.e. how the population changes in time following the intervention compared to the natural course of the mosquito population. We assume that a proportion ρ of mosquitoes is killed on the day of ULV-spraying, t_{ULV}, and we explore values of 10%, 20%, …, 100%. Following the intervention, new adults emerge from immature stages (which are not targeted by the ULV insecticide) according to the entomological model presented above. We computed the relative difference over time $\Delta_\rho(t)$ between the expected mosquito abundance without treatments and the actual abundance after t days. $\Delta_\rho(t)$ is a function of ρ and of the day of treatment t_{ULV}, which ranges from December 1, 2015 to June 30, 2016. By definition, $\Delta_\rho(0)=\rho$. After running the entomological model with several different values of ρ and t_{ULV}, we found that $\Delta_\rho(t)$ can be approximated by a negative exponential (Figure C). Higher values of ρ produce more lasting reduction effects. Treatments carried out during the earlier part of the study period (red lines) have a shorter impact on the mosquito dynamics since dead mosquitoes are more quickly replaced by newly emerged ones during the central, warmer months.

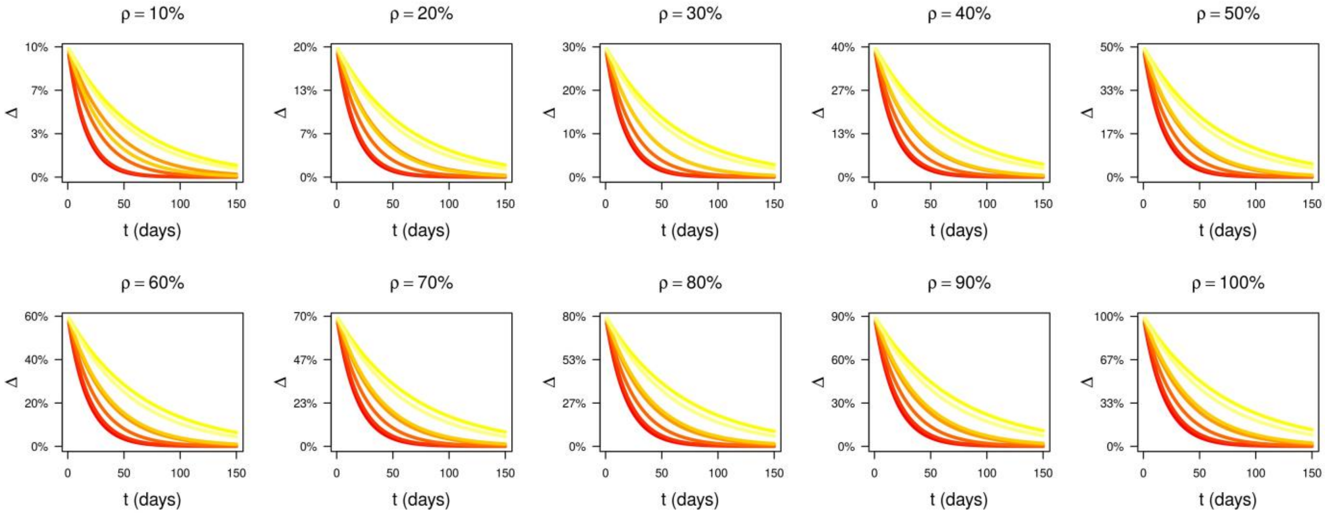

Figure C. Relative reduction $\Delta_\rho(t)$ of the adult mosquito population due to ULV-treatment. Different lines show the curve of $\Delta_\rho(t)$ for different times of treatment implementation, i.e. between December 1, 2015 (dark red) to June 30, 2016 (light yellow). X-axis: number of days after ULV-treatment.

Spatial DENV transmission model

For each cell $i \in N$, where N indicates the set of the 9919 geographic cells (100m x 100m) of the study area and cell i is located at coordinates (x_i, y_i), the DENV transmission dynamics is modelled according to the following ODE:

$$H'_{S_i} = -\frac{bp_{MH}}{H_i}\sum_{j\in U_i} M_{I_j}\phi(j,i)$$
$$H'_{E_i} = \frac{bp_{MH}}{H_i}\sum_{j\in U_i} M_{I_j}\phi(j,i) - \delta_H H_{E_i}$$
$$H'_{I_i} = \delta_H p_s H_{E_i} - \gamma_H H_{I_i}$$
$$H'_{As_i} = \delta_H(1-p_s)H_{E_i} - \gamma_H H_{As_i}$$
$$H'_{R_i} = \gamma_H\left(H_{I_i} + H_{As_i}\right)$$
$$M'_{S_i} = \Gamma_{M_i} - b\psi p_{HM}\sum_{j\in U_i}\left(\phi(j,i)\frac{H_{I_j} + a\,H_{As_j}}{H_j}\right)M_{S_i} - \mu_A M_{S_i}$$
$$M'_{E_i} = b\psi p_{HM}\sum_{j\in U_i}\left(\phi(j,i)\,\frac{H_{I_j} + a\,H_{As_j}}{H_j}\right)M_{S_i} - \delta_M M_{E_i} - \mu_A M_{E_i}$$
$$M'_{I_i} = \delta_M M_{E_i} - \mu_A M_{I_i}$$

Where

- $H_{S_i}, H_{E_i}, H_{I_i}, H_{As_i}, H_{R_i}$ are the number of susceptible, exposed, viraemic symptomatic, viraemic asymptomatic and removed individuals respectively in cell i. H_i is the total number of humans of cell i;
- $M_{S_i}, M_{E_i}, M_{I_i}$ are the number of susceptible, exposed and infectious mosquitoes respectively in cell i;
- a is the relative transmissibility of asymptomatic individuals compared to symptomatic patients, assumed to be 0 in the baseline and 0.5 and 1 in a sensitivity analysis (Ferguson et al. 2016);
- $\Gamma_{M_i}(t)$ represents the number of new susceptible mosquitoes emerging in cell i, at day t such that the total number of mosquitoes $\tilde{Q}(t,x_i,y_i) = M_{S_i}(t) + M_{E_i}(t) + M_{I_i}(t)$ matches $Q(t, x_i, y_i)$, the number of adult mosquitoes predicted for cell i at day t. Mathematically, it is defined as
$$\Gamma_{M_i}(t) = \begin{cases} Q(t,x_i,y_i) - \tilde{Q}(t,x_i,y_i), & Q(t,x_i,y_i) \geq \tilde{Q}(t,x_i,y_i) \\ 0\quad, & Q(t,x_i,y_i) < \tilde{Q}(t,x_i,y_i) \end{cases}$$
- U_i is the set given by i and its surrounding cells. $\phi(j,i)$ is the contribution of cell j to the force of infection exerted on cell i, with $\sum_{j\in U_i}\phi(j,i) = 1$, computed according to a previously estimated distance-dependent kernel function for dengue transmission (Guzzetta et al. 2018) (see Figure D). Overall, $\phi(j,i)$ represents the relative probability of a human in cell i to receive a bite from a mosquito in cell j;
- ψ is a scaling factor for the force of infection;
- μ_A is the temperature-dependent natural death rate of mosquitoes.
- All other parameters and their values are reported in Table B.

We implemented a time-discrete stochastic version of the above-described model, with time–step $\Delta t = 1$ day. Precisely, for each cell the model is a Markov chain whose states represent the integer number of mosquitoes and individuals in all epidemiological stages. Transition probabilities are Poisson-distributed with means given by the rates shown in the equations.

According to the analysis carried out in (Guzzetta et al. 2018), Z=76 symptomatic importations occurred in the considered area and period, generating clusters with sizes Ω_i that were approximately exponentially distributed

with an average $\overline{\Omega}$ of 10.62 (95%CI 6.7-14.5). We simulated the clusters generated by such importations, i.e. with the same combination (x_0, y_0, t_0) of the 76 index cases identified in (Guzzetta et al. 2018), with all possible combinations obtained by running the scaling factor for the force of infection ψ between 0 and 1 with step 0.01 and ρ between 0 and 100% with step 10%, assuming only symptomatic individuals transmit the virus (a=0). Then, we computed the likelihood L that the observed cluster sizes were extracted from an exponential distribution with average given by the model predicted average cluster size, $\overline{\Omega}_{\mathrm{M}}(\rho, \psi)$

$$\mathrm{L} = \left(\frac{1}{\overline{\Omega}_{\mathrm{M}}(\rho, \psi)}\right)^{\mathrm{z}} \cdot \exp\left(-\frac{1}{\overline{\Omega}_{\mathrm{M}}(\rho, \psi)} \cdot \sum_{\mathrm{j}=1}^{\mathrm{z}} \Omega_{\mathrm{j}}\right).$$

Using a maximum likelihood approach, we obtain ψ=0.57 and ρ=40%. Figure E shows that the resulting distribution of cluster sizes in the model replicates well the distribution observed in (Guzzetta et al. 2018) (χ^2=2.56, df=4, p-value=0.63). The simulated average cluster size $\overline{\Omega}_{\mathrm{M}}(\rho, \psi)$ is 10.61, very close to $\overline{\Omega}$. For the scenario with no vector control intervention, we run the model with the same ψ and ρ=0%.

**Parameter**	**Explanation**	**Value**	**Source**
δ_M	Mosquito incubation period	$\dfrac{0.003359 \cdot \frac{T_k}{298} \cdot \exp\left(\frac{15000}{R} \cdot \left(\frac{1}{298} - \frac{1}{T_k}\right)\right)}{1 + \exp\left(6.203 \cdot \frac{10^{21}}{1.987} \cdot \left(-\frac{1}{2.176 \cdot 10^{30}} - \frac{1}{T_k}\right)\right)}$	Lourenco & Recker 2014
p_{HM}	Human-to-mosquito transmission probabilities for symptomatic individuals	$0.001044 \cdot T \cdot (T - 12.286) \cdot (32.461 - T)^{\frac{1}{2}}$	Lourenco & Recker 2014
p_{MH}	Mosquito-to-human transmission probabilities	$0.0729 \cdot T - 0.97$	Lourenco & Recker 2014
δ_H^{-1}	Human incubation period	6 days	Chan & Johansson 2012
γ_H^{-1}	Human infectious period	4 days	Lourenco & Recker 2014
b	Daily mosquito biting rate	0.051 bites / mosquito / day	Goindin et al. 2015
p_S	Proportion of symptomatic infections	0.45	Ferguson et al. 2016

Table B. Parameters of the DENV transmission model. T and T_K denote the temperature in Celsius and Kelvin respectively.

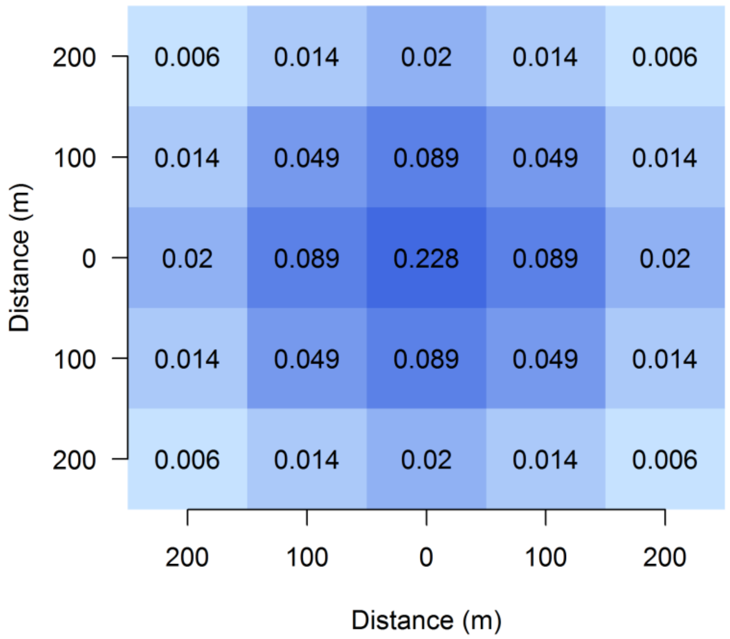

Figure D. Distance-dependent transmission kernel, encoded as the weights ϕ of cells in U_i.

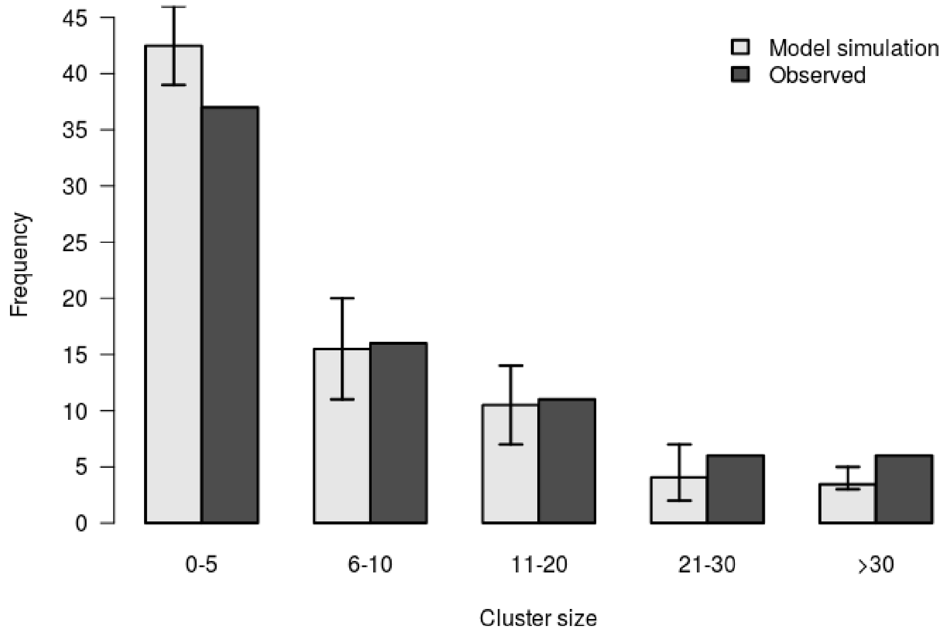

Figure E. Distribution of the cluster size occurred in the study period and area according to (Guzzetta et al. 2018) and as computed with the model with ρ=40% and ψ=0.57 (light and dark boxes respectively). Lines represent 95% confidence intervals.

Supplementary results

Effect of treatment on mosquito capture data

We compared mosquito captures from traps in the proximity of a treatment (e.g. within a radius R_1 from the case triggering the intervention) to corresponding captures collected at the same time from nearby surrounding areas (e.g. at a distance between R_1 and R_2 from the triggering case). We did not find a significant (Student t-test) difference in captures between treated and untreated areas under different combination of R_1 and R_2; non-significant differences were also found by comparing mosquito captures in the week preceding or following the treatment. We attributed this result to two factors related to data collection, combined with the weak effectiveness of treatment itself. First, capture data came from sticky traps that were inspected once per week: therefore, captures might reflect the full effect of treatment only when the interventions were done exactly on the first day of the capture week. Restricting the analysis to these cases only did not provide sufficient statistical power; on the other hand, even in these cases, mosquito populations would be able to partially recover within the capture week, given the exponential decay of the treatment effect (see Figure C). Secondly, sticky traps have a limited capture rate, so that the number of weekly captures per trap is very often of a few moquitoes; such small numbers, combined with the coarse temporal resolution of captures and the small ULV-induced mosquito mortality, make it difficult to detect a significant effect of treatment from capture data.

Full results from sensitivity analyses

Figure F shows the full range of reductions in symptomatic DENV cases that can be obtained with different combinations of the intervention radius r, and of the treatment delay τ with different values of ρ. Specifically, Figure F-a shows the effectiveness of different ρ and r values after fixing τ=14 days; similarly, Figure F-b shows the effectiveness of different ρ and τ values after fixing r=200m.

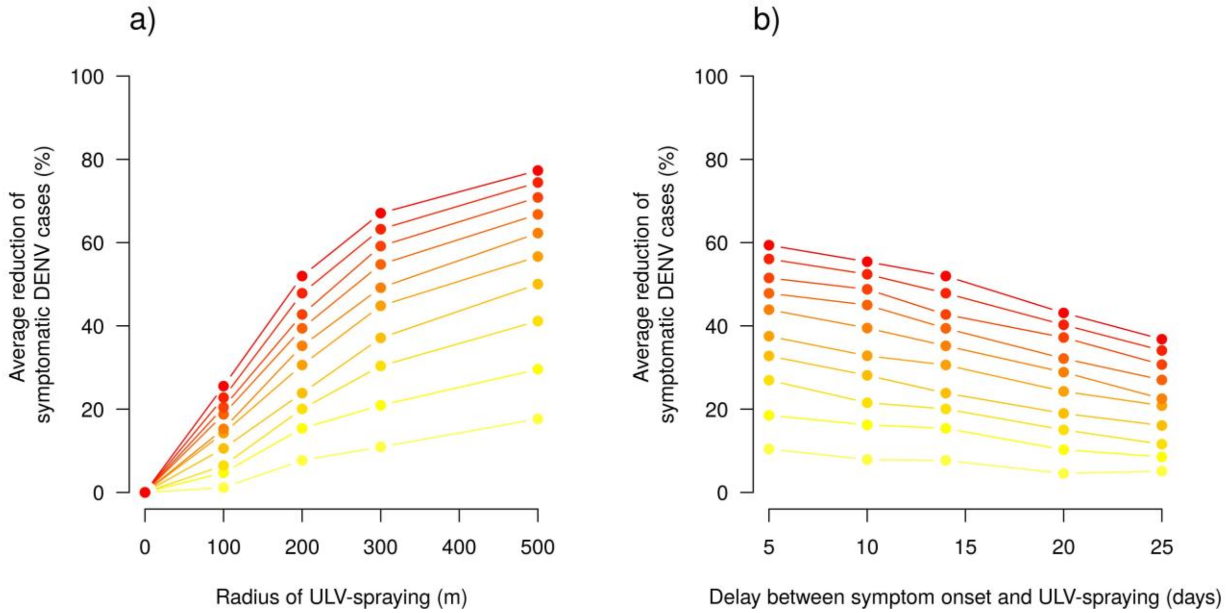

Figure F. Average percentage of avoided DENV symptomatic cases according to different vector control efficacies (from ρ=10%, light yellow, to ρ=100%, dark red) and different treated areas (panel a, τ=14 days) and average delays between symptom onset and intervention (panel b, *r*=200m).

Additionally, we investigated how different combinations of intervention protocol parameters (*r* and τ) would affect the reduction of DENV cases under the baseline value of the ULV induced mortality of 40%. As shown in Figure G, it is clear that the radius of the treated area is the most crucial parameter. By widening the treated area and decreasing the delay between symptom onset and intervention, authorities might avoid up to 58.8% DENV infections on average (*r*=500m and τ=5 days).

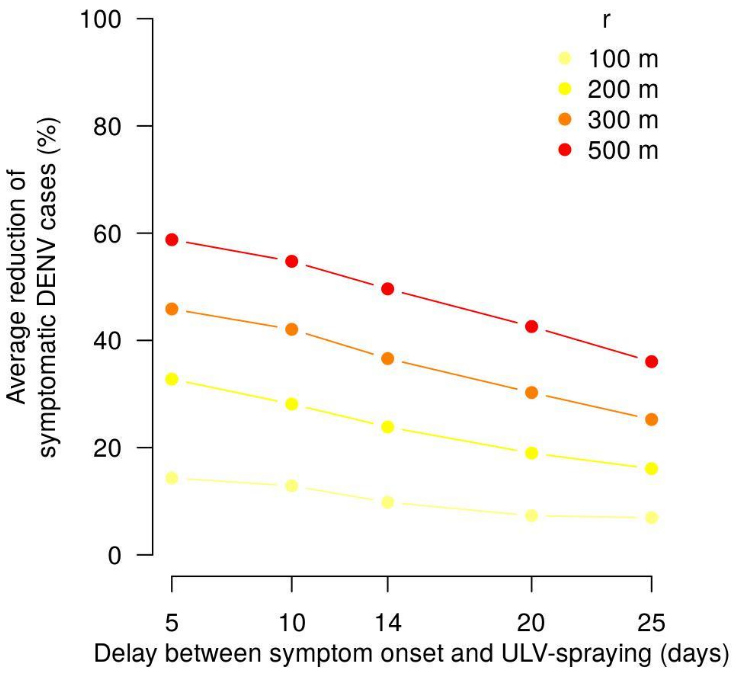

Figure G. Average percentage of avoided DENV symptomatic cases according to different treated areas (from *r*=100m, light yellow, to *r*=500m, dark red) and average delays between symptom onset and intervention with ρ=40%.

Transmission by asymptomatic individuals

In this section, we re-analyze the model under the assumption that asymptomatic humans can transmit DENV to mosquitoes with two different rates: i) at a halved rate than symptomatic individuals, i.e. *a*=0.5, similarly to the analysis performed in (Ferguson et al. 2016); ii) at the same rate than symptomatic individuals, i.e. *a*=1.

i) Halved asymptomatic transmission (a=0.5)

Using the same maximum-likelihood procedure deployed in the case *a*=0, we estimated *ψ*=0.35 and ρ=40%. In this scenario, such parameter combination yields an average cluster size of 10.6 for the 76 importations.

The resulting effectiveness of control interventions, considering ρ=40%, *r*=200m and τ=14 days, is comparable to the one estimated by assuming only symptomatic individuals can transmit the virus. In fact, the average cluster size reduction is 23.3% (95%CI: 15.1-31.4%), comparable to 23.9% found in the main analysis (see Figure H-a). This effectiveness is higher if the importation is symptomatic and triggers treatment, whereas it is much lower in the case of asymptomatic importations (see Figure H-b,c). In this latter case, only subsequent autochthonous symptomatic infections can be detected, thus interventions are implemented at least one infectious generation later compared to clusters where treatment was started for the index case. On the other hand, asymptomatic importations produce smaller clusters (8.9 symptomatic infections on average without control interventions). Changes in the proportion of mosquitoes killed by ULV resulted in proportional reductions of secondary symptomatic DENV cases similarly to what presented in the main text as well, up to a maximum of 47.9% when all existing mosquitoes are killed by treatment under current intervention protocols (Figure H-d).

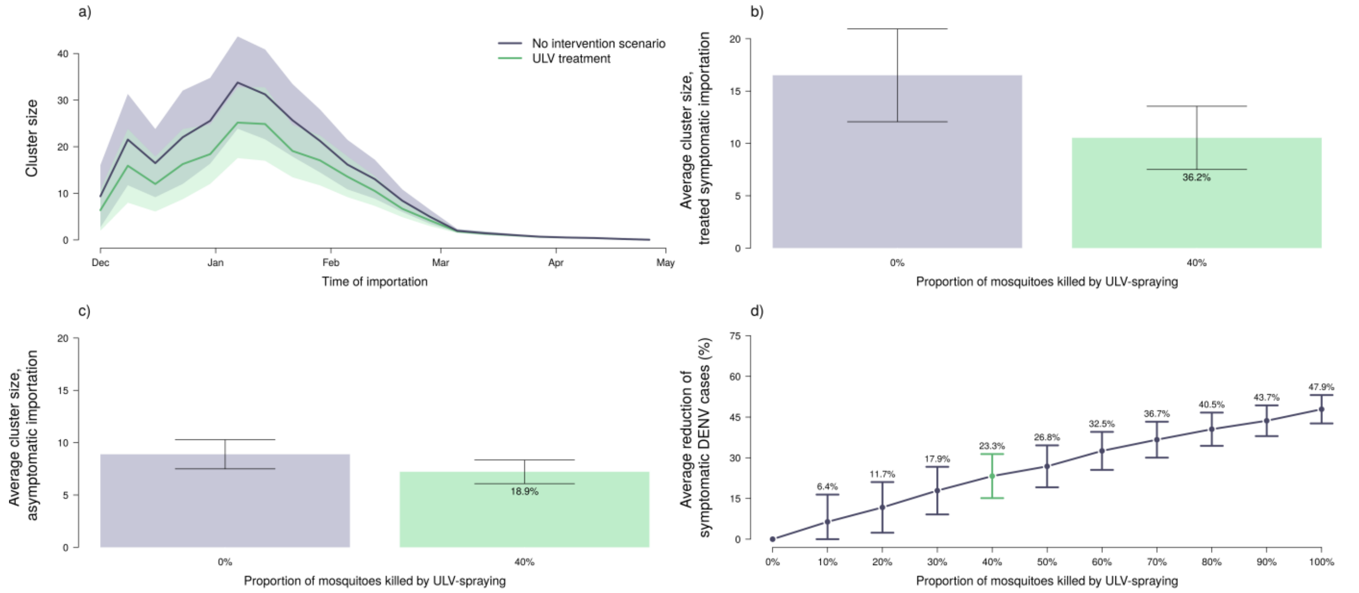

Figure H. Effect of control interventions on cluster size assuming asymptomatic individuals can transmit the virus at a halved rate. a) Cluster size over time; b) average cluster size when treatment is triggered by the index case; c) average cluster size for asymptomatic importations; d) average percentage of avoided DENV symptomatic cases by proportion of mosquitoes killed by ULV spraying (τ=14 days, r=200m). Solid lines: average; shaded area: 95% credible interval. Numbers within bars indicate the average percentage of avoided DENV symptomatic cases.

ii) Full symptomatic transmission (a=1)

We also analyzed an extreme assumption scenario in which asymptomatic individuals are as viremic as symptomatic ones and thus transmit the infection to mosquitoes with the same rate. Following the same re-calibration procedure carried out for a=0 and a=0.5, we estimated ψ to be 0.27 and ρ=40%.

The resulting average effectiveness of control interventions, considering ρ=40%, r=200m and τ=14 days, is 22.1% (95%CI: 13.9-30.2%), again very close to the 23.9% found in the main analysis (see Figure I). This effectiveness is higher if the importation is symptomatic and triggers treatment. In the case of asymptomatic importation, the average cluster size in absence of control interventions is equal to the value for symptomatic index cases. The different percentages of avoided DENV cases found assuming the importation is symptomatic or asymptomatic are similar to the ones found in the case a=0.5.

Changes in the proportion of mosquitoes killed by ULV resulted in proportional reductions of secondary symptomatic DENV cases similarly to what presented in the main text as well, up to a maximum of 46% when all existing mosquitoes are killed by treatment under current intervention protocols (Figure I-d).

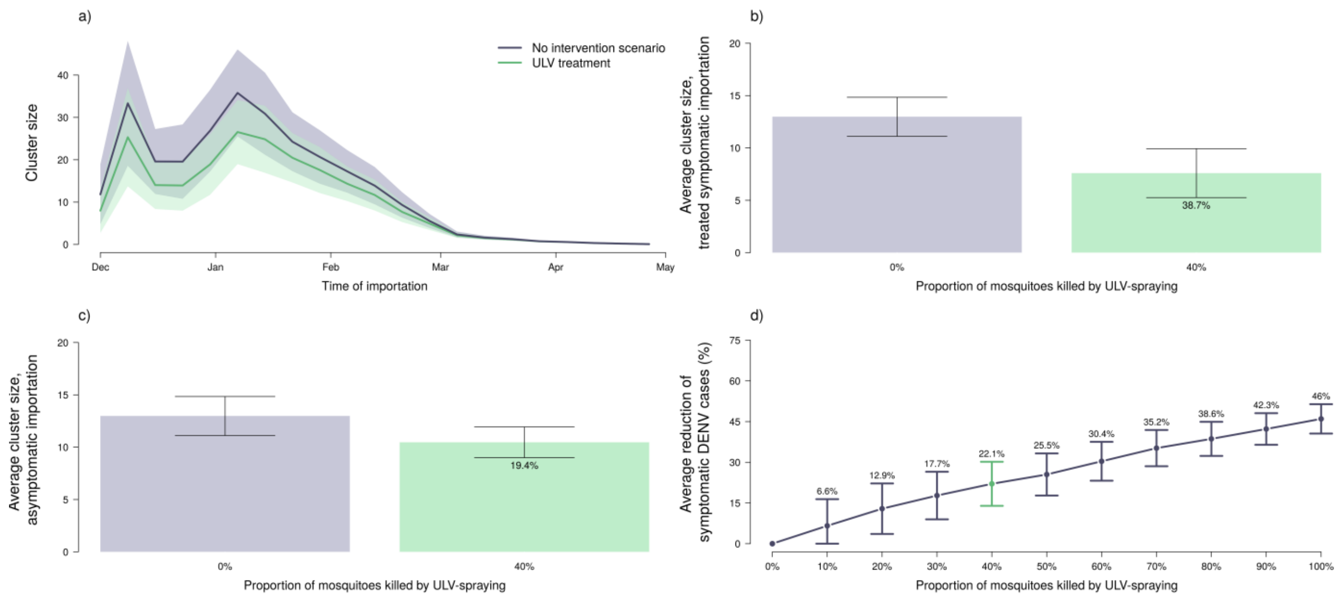

Figure I. Effect of control interventions on cluster size assuming asymptomatic individuals can transmit the virus as symptomatic individuals. a) Cluster size over time; b) average cluster size when treatment is triggered by the index case; c) average cluster size for asymptomatic importations; d) average percentage of avoided DENV symptomatic cases by proportion of mosquitoes killed by ULV spraying (τ=14 days, *r*=200m). Solid lines: average; shaded area: 95% credible interval. Numbers within bars indicate the average percentage of avoided DENV symptomatic cases.

Finally, we can note that Figures H-d, I-d and 5c show very similar patterns, meaning that the estimated percentage of avoided DENV infections does not depend on the assumptions on virus transmissibility by asymptomatic individuals.

Systematic treatment of cases

In this section, we re-analyze the model under the assumption that confirmed cases always trigger an ULV treatment, i.e. $q_I = q_A = 1$.

The resulting effectiveness of control interventions, under baseline values of ρ=40%, *r*=200m and τ=14 days, is higher, with an average reduction in cluster size of 38.6% (95%CI: 33.6-43.7%) compared to 23.9% in the main analysis (see Figure J). We can note that such efficacy is very close the one obtained with *r*=300m and q_I and q_A as observed (37.1%, see Figure 4a in the main text), so carrying out the control treatment with the current protocol but higher radius would yield to the same reduction as performing it for each confirmed case.

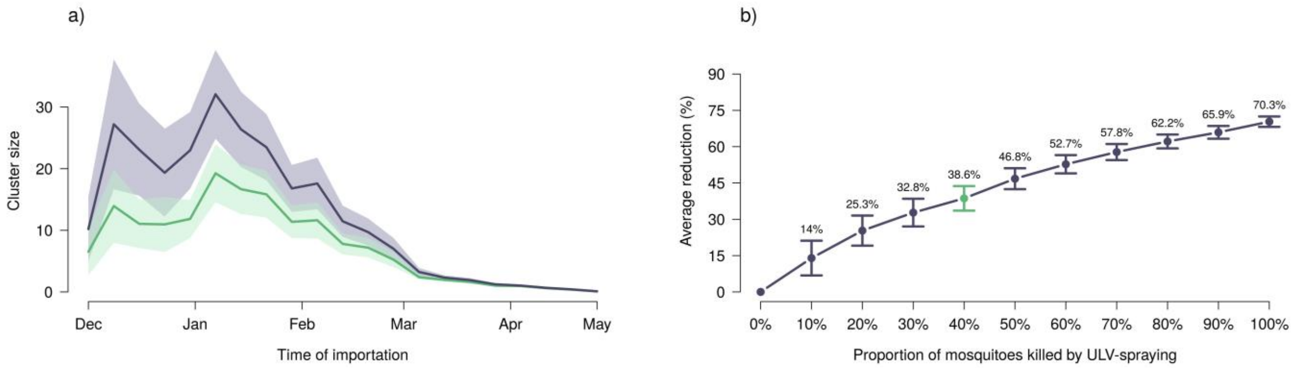

Figure J. Effect of control interventions on cluster size assuming ULV treatments are always implemented after confirmation of a case. a) Cluster size over time; b) Average percentage of avoided DENV symptomatic cases by proportion of mosquitoes killed by ULV spraying. In all panels, green lines show the baseline effectiveness computed at ρ=30%, *r*=200m and τ=14 days.

References

Chan M, Johansson MA. 2012. The Incubation Periods of Dengue Viruses. PloS One 7(11):e50972.

Ferguson NM, Rodríguez-Barraquer I, Dorigatti I, Mier-y-Teran-Romero L, Laydon DJ, Cummings DA. 2016. Benefits and risks of the Sanofi-Pasteur dengue vaccine: Modeling optimal deployment. Science 353(6303):1033-6.

Goindin D, Delannay C, Ramdini C, Gustave J, Fouque F. 2015. Parity and Longevity of Aedes Aegypti According to Temperatures in Controlled Conditions and Consequences on Dengue Transmission Risks. PloS One 10(8):e0135489.

Gubler DJ 2011. Dengue, Urbanization and Globalization: The Unholy Trinity of the 21st Century. Tropical Medicine and Health 39 (4 Suppl):3–11.

Guzzetta G, Marques Toledo CA, Rosà R, Teixeira M, Merler S. 2018. Quantifying the spatial spread of dengue in a non-endemic Brazilian metropolis via transmission chain reconstruction. Nature Communications 9:2837.

Lourenço J, Recker M. 2014. The 2012 Madeira Dengue Outbreak: Epidemiological Determinants and Future Epidemic Potential. PLoS Neglected Tropical Diseases 8(8):e3083.

Maciel-de-Freitas R, Loureno-de-Oliveira R. 2009. Presumed Unconstrained Dispersal of Aedes Aegypti in the City of Rio de Janeiro, Brazil. Revista De Saude Publica 43(1):8–12.

Otero M, Solari HG, Schweigmann N. 2006. A Stochastic Population Dynamics Model for Aedes Aegypti: Formulation and Application to a City with Temperate Climate. Bulletin of Mathematical Biology 68(8):1945–74.
